# Targeting mitochondrial complex I using BAY 87-2243 reduces melanoma tumor growth

**DOI:** 10.1186/s40170-015-0138-0

**Published:** 2015-10-20

**Authors:** Laura Schöckel, Andrea Glasauer, Farhan Basit, Katharina Bitschar, Hoa Truong, Gerrit Erdmann, Carolyn Algire, Andrea Hägebarth, Peter HGM Willems, Charlotte Kopitz, Werner JH Koopman, Mélanie Héroult

**Affiliations:** BPH, GDD, Global Therapeutic Research Group Oncology II, Bayer Pharma AG, Müllerstraße 178, 13353 Berlin, Germany; Department of Biochemistry, Radboud Institute for Molecular Life Science (RIMLS), Radboud University Medical Centre (RUMC), Nijmegen, The Netherlands; Bayer AG Innovation Strategy, Kaiser Wilhelm Allee 1, 51368 Leverkusen, Germany

**Keywords:** Anti-tumor efficacy, Mitochondrial complex I, Reactive oxygen species (ROS), Oxidative phosphorylation (OXPHOS), Small molecule inhibitor, BRAF mutant melanoma, Cancer metabolism

## Abstract

**Background:**

Numerous studies have demonstrated that functional mitochondria are required for tumorigenesis, suggesting that mitochondrial oxidative phosphorylation (OXPHOS) might be a potential target for cancer therapy. In this study, we investigated the effects of BAY 87-2243, a small molecule that inhibits the first OXPHOS enzyme (complex I), in melanoma in vitro and in vivo.

**Results:**

BAY 87-2243 decreased mitochondrial oxygen consumption and induced partial depolarization of the mitochondrial membrane potential. This was associated with increased reactive oxygen species (ROS) levels, lowering of total cellular ATP levels, activation of AMP-activated protein kinase (AMPK), and reduced cell viability. The latter was rescued by the antioxidant vitamin E and high extracellular glucose levels (25 mM), indicating the involvement of ROS-induced cell death and a dependence on glycolysis for cell survival upon BAY 87-2243 treatment. BAY 87-2243 significantly reduced tumor growth in various BRAF mutant melanoma mouse xenografts and patient-derived melanoma mouse models. Furthermore, we provide evidence that inhibition of mutated BRAF using the specific small molecule inhibitor vemurafenib increased the OXPHOS dependency of BRAF mutant melanoma cells. As a consequence, the combination of both inhibitors augmented the anti-tumor effect of BAY 87-2243 in a BRAF mutant melanoma mouse xenograft model.

**Conclusions:**

Taken together, our results suggest that complex I inhibition has potential clinical applications as a single agent in melanoma and also might be efficacious in combination with BRAF inhibitors in the treatment of patients with BRAF mutant melanoma.

**Electronic supplementary material:**

The online version of this article (doi:10.1186/s40170-015-0138-0) contains supplementary material, which is available to authorized users.

## Background

It has been well established that cancer cells are characterized by an altered metabolism compared to normal cells. In fact, cancer cells reprogram their metabolism to meet increased energetic and anabolic needs, including those for ATP, reducing equivalents (e.g., NADPH), lipids, proteins, and nucleotides. The metabolic adaptation of cancer cells enables rapid cell proliferation and sustained cell survival even in extreme tumor microenvironments exhibiting hypoxia and nutrient deprivation [1]. Historically, mitochondrial metabolism and oxidative phosphorylation (OXPHOS) were considered to be expendable for the metabolic demands of rapidly dividing cells [2, 3]. This classical observation, made by Otto Warburg in the 1920s, states that cancer cells convert glucose into lactate even in the presence of sufficiently high oxygen levels. It is now appreciated that mitochondrial metabolism is also pivotal for the generation of building blocks required for cancer cell proliferation and that the “Warburg effect” (also known as aerobic glycolysis) does not fully compensate for defects in mitochondrial ATP production due to damaged mitochondria as originally proposed. In fact, the majority of cancer cells display functional mitochondria and are able to generate ATP through mitochondrial metabolism fueled by fatty acids and amino acids, such as glutamine [4–9]. Additionally, cancer cells also require mitochondrial oxidative metabolism to maintain their redox balance. Depending on the cellular context, mitochondria substantially contribute to the generation of cellular reactive oxygen species (ROS). Under physiological conditions, ROS formation at complex I and III of the mitochondrial electron transport chain is a natural by-product of mitochondrial ATP generation [10, 11] and occurs due to the incomplete reduction of molecular oxygen. Specifically in cancer cells, ROS appear to play a causal signaling role in tumor development and progression by inducing DNA mutations, genetic instability, and also pro-tumorigenic signaling [12]. When ROS production exceeds the capacity of intracellular ROS-detoxifying systems, cell-toxic oxidative stress is induced. This explains why cancer cells have to tightly control the balance between ROS generation and scavenging in order to remain within the pro-tumorigenic range of ROS levels [13]. In this sense, cancer cells display an increased ROS scavenging capacity that prevents ROS levels from reaching cytotoxic levels incompatible with growth [14, 15].

Given the important role of OXPHOS in mitochondrial and cell metabolism, its targeting may be useful in cancer therapy development. The first OXPHOS complex, complex I, constitutes the entry point of electrons and is a known site of ROS production [16]. Interestingly, evidence was provided showing that complex I inhibition by the anti-diabetic biguanide metformin inhibits tumorigenesis in vitro and in vivo [17–21]. Similarly, the biguanide phenformin might exert its anti-tumor effects by inhibiting complex I [22]. However, metformin and phenformin were used at very high concentrations to exert their anti-tumor efficacy, with phenformin also exerting an increased risk of lactic acidosis [23, 24].

In this study, we investigated the effects of BAY 87-2243, a potent and selective small molecule inhibitor of mitochondrial complex I, on mitochondrial function and anti-tumor activity. BAY 87-2243 has been shown to inhibit hypoxia-inducible factor (HIF) gene activation and displayed anti-tumor activity in an in vivo non-small cell lung cancer xenograft model [25]. However, the signaling mechanism by which BAY 87-2243 induced anti-tumorigenic effects is still unclear, and it has not been tested in other cancer models. Using BRAF mutant melanoma as a model system, we here report that BAY 87-2243-mediated complex I inhibition induced melanoma cell death in vitro and reduced melanoma tumor growth in various mouse models in vivo. Our results suggest that this effect is mediated through BAY 87-2243-induced stimulation of mitochondrial ROS production, leading to oxidative damage and subsequent cell death. Interestingly, we observed that BAY 87-2243 displayed increased anti-tumor efficacy compared to single agent treatment when combined with the small molecule mutant BRAF inhibitor vemurafenib in nude mice bearing BRAF mutant melanoma xenografts.

## Methods

### Animal studies and cell cultures

Tumor xenograft experiments were carried out on female immune-deficient mice in full accordance with the Interdisciplinary Principles and Guidelines for the Use of Animals in Research, Marketing and Education issued by the New York Academy of Sciences’ Ad Hoc Committee on Animal Research. Human melanoma SK-MEL-28 and G-361 cells were subcutaneously inoculated in athymic *nu*/*nu* mice (28–32 g, aged 7–8 weeks, Janvier) and Balbc/nude (18–25 g, aged 5–6 weeks, Janvier) mice, respectively. A-375 and LOX-IMVI melanoma cells were inoculated in scid (scid/scid) mice (20–25 g, aged 15–17 weeks, Charles River). The melanoma xenograft mouse model was established by subcutaneous injection into the right flank with 0.1 mL SK-MEL-28 cells (3 × 10E6) mixed 1:1 with Matrigel or 0.1 mL A-375 cells (1.5 × 10E6) or LOX-IMVI cells (1.5 × 10E6) mixed 1:1 with Matrigel or 5 × 10E6 G-361 cells in 100 % Matrigel (Becton Dickinson). Mice were randomized into control and treatment groups when tumors reached a size of more than 50 mm^2^. Treatment with vemurafenib (20 mg/kg/twice daily) or BAY 87-2243 (9 mg/kg/day) was administered by oral gavage in Ethanol/Solutol/Water (10/40/50). Body weight was monitored as a measure for treatment related, acute toxicity. Tumor areas (measured by caliper) were calculated according to the formula width × length. The human melanoma cell lines A-375, G-361, SK-MEL-5, SK-MEL-28, LOX-IMVI, SK-MEL-2, IPC-298, CHL-1, and Colo-792 were obtained from American Type Culture Collection (ATCC), grown at 37 °C and 5 % CO_2_. All cell lines were routinely grown in standard medium recommended by ATCC and supplemented with 10 % (v/v) fetal calf serum (FCS, Life technologies). When not stated differently, all experiments were carried out in phenol-red-free and pyruvate-free DMEM assay medium containing 5 mM glucose (Sigma), 2 mM GlutaMAX (Gibco), 5 % dialyzed FCS (Gibco).

### Western blot analysis

Melanoma cell lines (A-375, G-361, SK-MEL-5, SK-MEL-28) were grown to 80 % confluency and incubated with BAY 87-2243 (10 nM) or BAY 87-2243 (10 nM) in combination with either vitamin E (25 μM) or NAC (5 mM) for 8 or 16 h, whereas control samples were treated with an equal volume of DMSO. Cells were lysed in 100 μl RIPA lysis buffer (Roche) supplemented with complete protease inhibitor cocktail (Roche). Lysates were clarified by centrifugation (13,200 g, 15 min, 4 °C). The supernatant was transferred to a new tube, and protein levels were quantitated using the BCA method (Thermo Fischer). Using SDS-PAGE (Nu-PAGE 4–12 % Bis-Tris protein gels) and Western blotting, 30–50 μg total protein were analyzed with the following antibodies: anti-phospho-AMP-activated protein kinase (AMPK) (Thr172) (Cell Signaling, #2531), anti-AMPK (Cell Signaling, #2532), anti-phospho-RAPTOR (Ser792) (Cell Signaling, #2083), anti-phospho-p38 (Thr180/Tyr182) (Cell Signaling, #4511), anti-p38 (Cell Signaling, #9212), anti-NRF2 (Novus biologicals, NB100-80011), anti-phospho-ERK1/2 (Thr202/Tyr204) (Cell Signaling, #4377), anti-ERK1/2 (Cell Signaling, #9102), anti-cleaved PARP (Asp214) (Cell Signaling, #9541), and anti-β-actin (Sigma), followed by secondary goat-anti-mouse (IRDye800CW) or secondary goat-anti-rabbit (IRDye680LT) antibodies. Antibody signals were detected and quantitated using a LI-COR instrument.

### Analysis of bioenergetics using the Seahorse XF96 extracellular flux analyzer

Extracellular flux analyses were performed using the Seahorse XF96 Extracellular Flux Analyzer (Seahorse Bioscience). To determine the effects of vemurafenib, XF96 tissue culture plates were seeded at 20,000 cells/well. When adherent cells were fully attached, cells were treated with either DMSO or vemurafenib (1 μM) for 72 h. Basal mitochondrial function and mitochondrial stress in response to BAY-872243 were measured by oxygen consumption rate (OCR) using the XF Mito Stress Test Kit (Seahorse Bioscience). The XF Mito Stress Test Kit reveals key parameters of mitochondrial function: basal respiration, ATP production, and respiratory capacity. Briefly, cells were seeded at equal densities (20,000–30,000 cells/well) into XF96 tissue culture plates. Cell media was changed 24 h after cell seeding into unbuffered Dulbecco’s modified Eagle’s medium (DMEM) containing 8.3 g/l DMEM (Sigma), 2 mM GlutaMAX (Invitrogen), 5 mM glucose (Sigma), 63.3 mM NaCl (Sigma), and adjusted pH to 7.4 with NaOH. The drug injection ports of the XF96 Assay Cartridge were loaded with assay reagents to a final concentration of 1 μM oligomycin, 0.5 μM FCCP, 1 μM rotenone, and 1 μM antimycin A. OCR and extracellular acidification rate (ECAR) were measured under basal conditions and/or after injection of various concentration of BAY 87-2243 following the XF Mito Stress Kit. All treatment conditions were analyzed with six replicates. The OCR values were normalized to cell numbers plated. To this end, the cells were stained using Cyquant (Life technologies) and fluorescence was measured using a microplate reader (Tecan) with excitation at 485 nm and emission detection at 530 nm.

### High-resolution respirometry

The culture medium was collected and the BRAF mutant melanoma cells were trypsinized, washed, and resuspended to approximately 2 × 10E6 cells per 2 ml in the collected culture medium and used to measure cellular oxygen consumption. Oxygen consumption was measured at 37 °C using polarographic oxygen sensors in a two-chamber Oxygraph (Oroboros Instruments, Innsbruck, Austria) as previously described [26]. Briefly, cells were first allowed to respire at basal level for 10 min and then inhibited acutely by a stepwise addition of increasing concentrations (0–100 nM) of BAY 87-2243 until respiration was maximally blocked. Basal (routine) respiration was set at 100 % to which all other data points were related.

### Cell viability, cell death, metabolic assays, and flow cytometry

Melanoma cells were seeded in 96 well plates in triplicates at a density of 3000–5000 cells/well and incubated in culture medium. The next day, the culture medium was exchanged to assay medium as described above and cells were treated with various concentrations of BAY-872243 or DMSO added to media using a dispenser (HP D300 digital dispenser). After 72 h, cell viability was measured using CellTiter-Fluor (CTF, Promega) according to the manufacturer’s protocol. Fluorescence was detected (excitation 380 nm, emission 505 nm) in a microplate reader (Tecan) after 30 min of incubation at 37 °C. IC50 values were calculated from triplicate experiments using GraphPad prism software, using curves from plots of fluorescence intensity vs. BAY 87-2243 in each cell line. For the analysis of time-dependent effects of BAY 87-2243, cell viability was assessed by crystal violet assay. Briefly, cells were seeded in 48 well plates, treated with BAY 87-2243 (10 nM) or DMSO as control and after incubation stained with crystal violet solution (0.5 % crystal violet, 30 % ethanol, 3 % formaldehyde). Then, plates were rinsed with water and crystal violet incorporated by the cells was re-solubilized in 1 % SDS solution. Absorbance at 550 nm was measured using a microplate spectrophotometer (Benchmark Plus, Biorad). Results are expressed as percentage of cell viability relative to untreated controls.

For measurements of cell death, 2 ml cells were seeded in six well plates at 200,000 cells/well. When adherent cells were fully attached, culture media was removed and replaced with assay media containing BAY 87-2243 (10 nM), vitamin E (25 μM), or DMSO as a control. Cell death was measured after 72 h using propidium iodine and flow cytometry.

Extracellular lactate was measured in assay medium of treated cells using Cobas 6000 c501 analyzer (Roche). Results were normalized to cell number.

ATP measurements were conducted using CTG (Promega) according to the manufacturer’s protocol.

For MitoTracker stainings, cells were seeded into 384 well imaging plates (cell carrier, Perkin Elmer) at 1000 cells/well in 30 μL and incubated over night at 37 °C and 5 % CO_2_. Then, cells were treated with vemurafenib (1 μM) or DMSO as control for 72 h. Mitotracker Green FM (Invitrogen) staining was carried out using manufacturer’s recommended protocols. The DNA dye Hoechst was added to the MitoTracker staining solution (in DMEM culture medium) to achieve a final concentration of 1 μg/ml Hoechst and 100 nM MitoTracker Green FM. Cells were then incubated for 30 min at 37 °C and 5 % CO_2_. Afterwards, staining solution was removed and 50 μl/well pre-warmed medium was added. The cells were then imaged with the Opera® spinning disk automated microscope (Perkin Elmer), imaging six wells per condition with sites/well using a 20× objective utilizing separate 405 nm and 488 nm laser excitation. Imaging data was subsequently analyzed using the MetaXpress Software (Version 5, Molecular Devices). Nuclei segmentation and mitochondrial staining quantification was performed on using built in detection routines. As measurement for mitochondrial mass, total mitochondria staining intensity per well was divided by the total count of nuclei per well. Data are represented as the mean ± SD normalized to relative cell number.

### ROS measurements

To measure intracellular ROS levels, 5 μM cell permeable dichlorofluorescein diacetate (CM-H_2_DCFDA) (Invitrogen) or 5 μM cell permeable MitoSOX (Life technologies) were used as fluorescent dyes. The non-fluorescent redox-reactive dye CM-H_2_DCFDA can get oxidized by intracellular ROS yielding the fluorescent probe CM-H_2_DCF, which is proportional to cytosolic ROS. MitoSOX describes the non-fluorescent dye hydroethidium which gets oxidized by superoxide radicals yielding the fluorescent ethidium product. An equal number of cells were seeded in six well plates, treated with various concentrations of BAY 87-2243 or BAY 87-2243 (10 nM) in combination with vitamin E (25 μM) or NAC (5 mM) as indicated or 16 h. Cells were incubated with phenol-red-free assay medium containing either CM-H_2_DCFDA or MitoSOX dye in for 30 min at 37 °C. Cells were then washed with PBS, trypsinized, resuspended in PBS, and immediately analyzed by flow cytometry.

### Biochemicals

BAY 87-2243 was developed by Bayer Pharma AG [25]. Vemurafenib was obtained from APAC Pharma (Charge CLEV11988-2-2 APAC lot APWJ20130901PLX). *N*-Acetyl-cysteine (NAC) and vitamin E (α-tocopherol) were obtained from Sigma.

### Statistics

GraphPad software was used for statistical analysis. Statistical analyses for the comparison of more than two groups were performed by one-way ANOVA, followed by a Sidak’s multiple comparison test (pairwise comparisons). A two-tailed *t* test was performed to compare between two groups (two-group comparisons). *P* < 0.05 was considered to indicate a statistically significant difference.

## Results

### Inhibition of mitochondrial complex I with BAY 87-2243 in melanoma cells induces cell death in vitro and reduces tumor growth in vivo

**Fig. 1:**
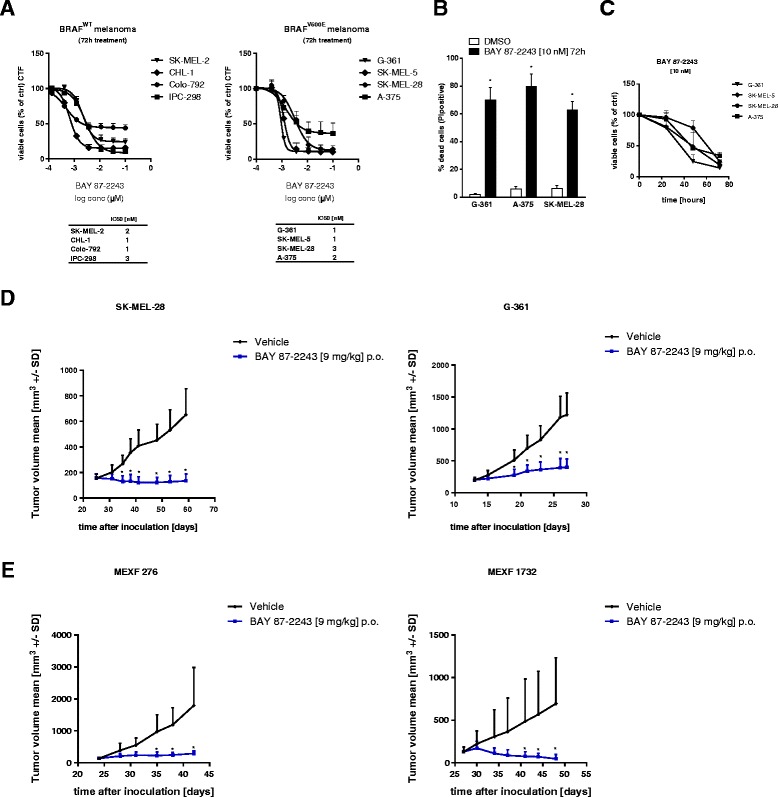
Inhibition of mitochondrial complex I with BAY 87-2243 in melanoma cells induces cell death in vitro and reduces tumor growth in vivo. **a** BRAF wild type and mutant melanoma cells were treated with various concentrations of BAY 87-2243. Cell viability was measured after 72 h. IC50 values were calculated using GraphPad prism (*n* = 4). **b** Melanoma cells were treated with BAY 87-2243 (10 nM). Cell death was measured after 72 h using propidium iodine (*n* = 4). **c** Melanoma cells were treated with BAY 87-2243 (10 nM). Cell viability was measured after 24, 48, and 72 h (*n* = 3). **d** Nude mice were xenotransplanted with either 3 × 10^6^ SK-MEL-28 (50 % matrigel) or 5 × 10^6^ G-361 (100 % matrigel) human melanoma cells (*n* = 10 per group). When tumor area reached around 50 mm^2^, mice were treated once a day with vehicle or 9 mg/kg BAY 87-2243 per oral gavage (p.o.). **e** Nude mice bearing patient-derived (MEXF 276, MEXF 1732, *n* = 8 per group) melanoma xenograft tumors were treated orally (p.o.), once a day with vehicle or 9 mg/kg BAY 87-2243. Data are represented as the mean ± SD. *p < 0.05

BAY 87-2243 has been shown to be a highly potent and selective inhibitor of mitochondrial complex I [25]. We demonstrate that BAY 87-2243-mediated complex I inhibition induced significant reduction of cell viability in a dose-dependent manner in several BRAF^V600E^ (G-361, SK-MEL-5, SK-MEL-28, and A-375) and also BRAF wild type (SK-MEL-2, IPC-298, CHL-1, and Colo-792) melanoma cell lines. BAY 87-2243 showed highly potent activity with an estimated IC50 in the one-digit nanomolar range after 3 days of treatment **(**Fig. 1a**)**. Using the same treatment protocol, BAY 87-2243 induced 70–80 % of cell death and reduced viability in a time-dependent manner (Fig. 1b, c).

To address whether BAY 87-2243-mediated complex I inhibition is efficient at reducing melanoma tumor growth in vivo, we tested the inhibitor in four BRAF mutant melanoma xenograft models using G-361, SK-MEL-28, A-375, and LOX-IMVI cancer cells. Upon subcutaneous melanoma cell injection, tumors were allowed to grow to around 50 mm^2^ before the mice were treated with vehicle or 9 mg/kg BAY 87-2243 (once a day) by oral gavage (p.o.). BAY 87-2243 induced a significant reduction in tumor size in all BRAF mutant melanoma xenografts, as well as a reduction in tumor weight without affecting body weight of the mice (Fig. 1d and Additional file 1: Figure S1A, B, C). Interestingly, BAY 87-2243 has stronger activity in the slow-growing melanoma xenografts (G-361 and SK-MEL-28). Also, BAY 87-2243 displayed a strong reduction in tumor growth in two patient-derived BRAF melanoma models (MEXF 276 and MEXF 1732) (Fig. 1e).

### Inhibition of mitochondrial complex I with BAY 87-2243 blocks OXPHOS and triggers glycolysis in melanoma cells

**Fig. 2:**
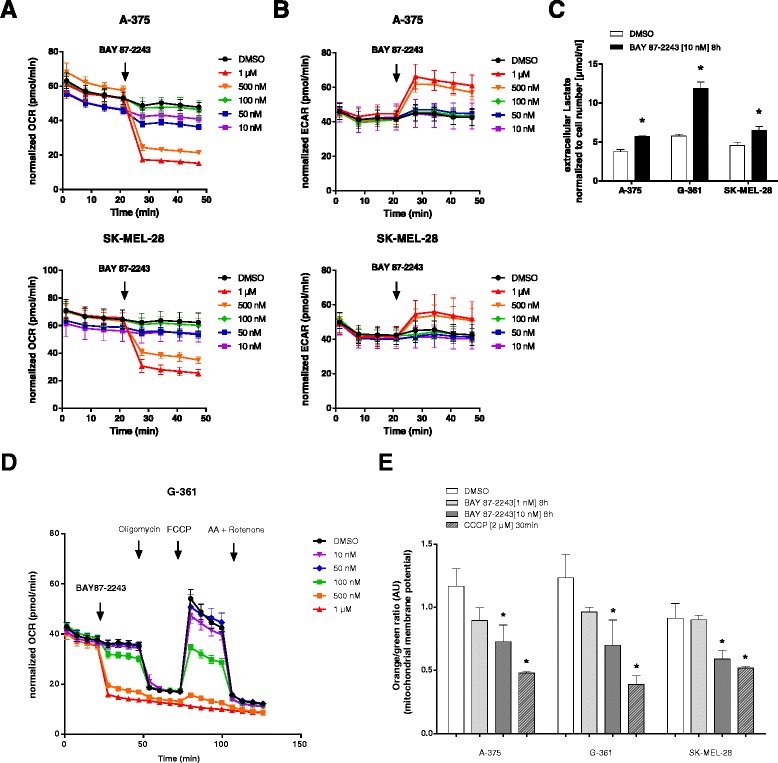
Inhibition of mitochondrial complex I with BAY 87-2243 blocks OXPHOS and triggers glycolysis in melanoma cells. **a** OCR was measured using Seahorse analyzer in BRAF mutant melanoma cells. BAY 87-2243 was injected (*black arrow*) in different concentrations (*n* = 6). **b** ECAR was measured using Seahorse analyzer in BRAF mutant melanoma cells. BAY 87-2243 was injected (*black arrow*) in different concentrations (*n* = 6). **c** Melanoma cells were treated with BAY 87-2243 (10 nM). Extracellular lactate was measured in cell culture supernatant after 8 h (*n* = 4). **d** OCR was measured using Seahorse analyzer in G-361 cells. BAY 87-2243 was injected (*black arrow*) in different concentrations followed by consecutive injections of oligomycin (1 μM), FCCP (0.5 μM), and antimycin A (1 μM)/rotenone (1 μM) (*n* = 6). **e** Melanoma cells were treated with different concentrations of BAY 87-2243. After 8 h, mitochondrial membrane potential was measured using Mito-ID assay (*n* = 3). CCCP (2 μM) was used as positive control. Data are represented as the mean ± SD. *p < 0.05

To investigate the mechanism by which BAY 87-2243-mediated mitochondrial complex I inhibition induced cell death and anti-tumor activity in melanoma cells, we first determined the direct effect of the inhibitor on mitochondrial oxygen consumption. Addition of BAY 87-2243 induced a dose-dependent reduction of basal OCR in A-375 and SK-MEL-28 cells (Fig. 2a). Using polarographic oxygen sensors a strong reduction in OCR was also already observed in the low nanomolar range matching the IC50 viability results (Fig. 1a and Additional file 2: Figure S2A). This indicates selective inhibition of mitochondrial complex I and rationalizes the use of minimal concentrations of BAY 87-2243 that maximally inhibit cell growth (Fig. 1a) and mitochondrial respiration (Additional file 2: Figure S2A) in all further experiments.

In the same cells, the ECAR of the cell culture media, an indirect readout of glycolysis, increased (Fig. 2b) which was also accompanied with a significant rise in extracellular lactate of BAY 87-2243-treated cells (Fig. 2c). This data indicates that BAY 87-2243 induced a gradual switch from OXPHOS to glycolysis demonstrated by the immediate drop in OCR and the time-dependent increase in the glycolytic product lactate (Additional file 2: Figure S2B). Detailed investigation of mitochondrial function showed that, in addition to decreasing basal OCR, BAY 87-2243 also resulted in diminished maximal respiratory capacity of the mitochondria, which was seen upon the addition of the mitochondrial uncoupler FCCP (Fig. 2d and Additional file 2: Figure S2C). Furthermore, BAY 87-2243 treatment induced a partial mitochondrial membrane depolarization in melanoma cells which can potentially lead to an energy crisis due to the lack in ATP production (Fig. 2e).

### Complex I inhibition using BAY 87-2243 reduces ATP levels and induces an energy crisis in melanoma cells

**Fig. 3:**
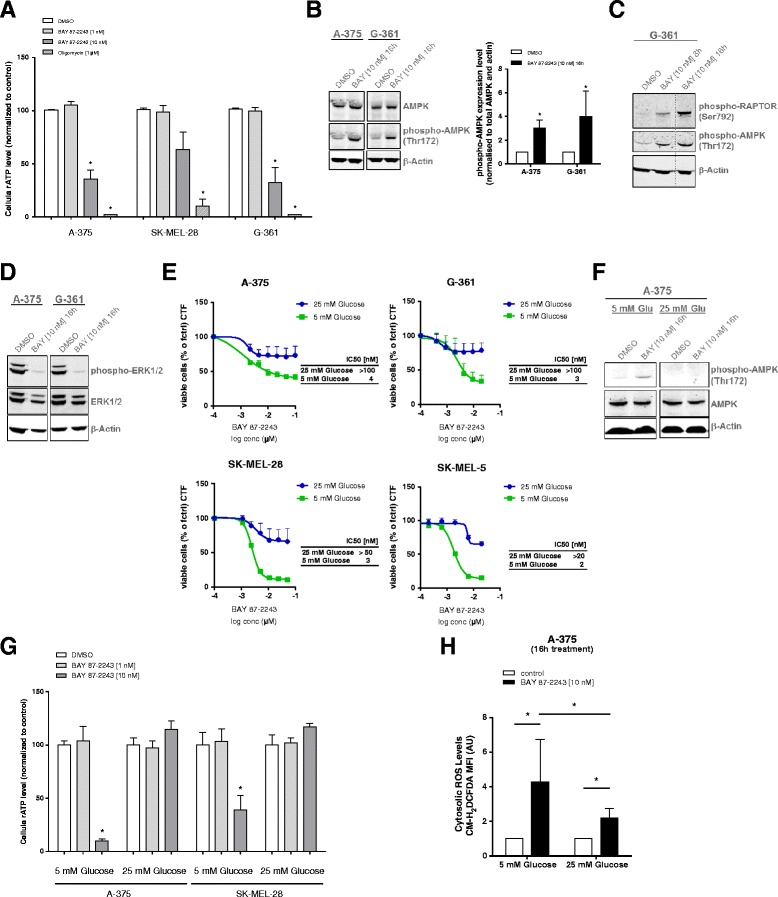
Complex I inhibition using BAY 87-2243 reduces ATP levels and induces an energy crisis in melanoma cells. **a** Melanoma cells were treated with different concentrations of BAY 87-2243 and ATP was measured after 8 h. Oligomycin (1 μM) served as positive control (*n* = 3). **b**, **c** Melanoma cells were treated with BAY 87-2243 (10 nM). Cell lysates were collected from treated cells (16 h) and probed with antibodies recognizing phospho-AMPK (Thr172), total AMPK, **c** phospho-RAPTOR (Ser792), **d** anti-phospho-ERK1/2 (Thr202/Tyr204), and anti-ERK1/2. Actin was used as a loading control. **e** Melanoma cells were treated with various concentrations of BAY 87-2243. Cell viability was measured in high (25 mM) and low (5 mM) glucose medium after 72 h (*n* = 3). IC50 values were calculated using GraphPad Prism. **f** A-375 cells were treated with BAY 87-2243 (10 nM) under low- (5 mM) and high (25 mM) glucose conditions. Cell lysates were collected from treated cells (16 h) and probed with antibodies recognizing phospho-AMPK (Thr172) and total AMPK. Actin was used as a loading control. **g** Melanoma cells were treated with BAY 87-2243 (10 nM) under low- (5 mM) and high (25 mM) glucose conditions and ATP was measured after 16 h (*n* = 3). **h** A-375 cells were treated with BAY 87-2243 (10 nM) under low- (5 mM) and high (25 mM) glucose conditions. Cytosolic ROS levels were measured using the ROS-reactive dye CM-H_2_DCFDA after 16 h (*n* = 3). Data are represented as the mean ± SD. *p < 0.05

To further address the mechanism behind the BAY 87-2243-induced anti-tumorigenic phenotype, cellular ATP levels were measured. This also allowed us to investigate the role of the observed changes in mitochondrial respiration and membrane polarization. We show that BAY 87-2243 caused a drastic, dose-dependent decrease of total cellular ATP levels in melanoma cell lines, 8 h after inhibitor treatment (Fig. 3a). In order to determine if this drop in ATP had an effect on metabolic signaling, we looked at the activity of AMPK upon BAY 87-2243 treatment, a critical energy sensor that is activated by low ATP levels. It has been shown that AMPK is activated in the liver in response to therapeutic doses of metformin and phenformin due to mitochondrial complex I inhibition [27]. Here, we found that BAY 87-2243 significantly induced phosphorylation and activation of AMPK in melanoma cells (Fig. 3b) which is consistent with the drop in ATP levels (Fig. 3a). BAY 87-2243 also activated RAPTOR, a well-defined downstream target of AMPK (Fig. 3c). These results suggest that BAY 87-2243 induces an energy crisis in melanoma cell lines, which causes AMPK and RAPTOR activation. Interestingly, BAY 87-2243-mediated complex I inhibition also reduced the activation of extracellular-signal-regulated kinase 1/2 (ERK1/2) (Fig. 3d). Keeping in mind that BAY 87-2243 appeared to stimulate glycolysis (Fig. 2 and Additional file 2: Figure S2) potentially leading to the depletion of extracellular glucose, we next cultured the melanoma cells in a medium with high glucose (25 mM). Under these conditions cell viability was rescued (Fig. 3e), AMPK was not activated (Fig. 3f) and cellular ATP content was not reduced in BAY 87-2243-treated melanoma cells (Fig. 3g). Taken together, these results suggest that melanoma cells require glucose-driven glycolysis for ATP production to survive in the presence of BAY 87-2243.

### BAY 87-2243-mediated complex I inhibition increases mitochondrial and cytosolic ROS levels resulting in ROS-mediated cell death

Next, we focused on already known outcomes of complex I inhibition and their potential role in causing the anti-tumorigenic phenotype upon BAY 87-2243 treatment. It is well established that mitochondrial complex I constitutes a major source of ROS which can be toxic to the cell at high levels. Studies have shown that the known complex I inhibitor rotenone significantly induces the production of high levels of mitochondrial ROS, leading to oxidative stress [28, 29]. Interestingly, here, we found that culturing BAY 87-2243-treated melanoma cells in excess glucose media (25 mM) did not only stabilize ATP levels (Fig. 3g) but also significantly decreased ROS levels (Fig. 3h).

**Fig. 4:**
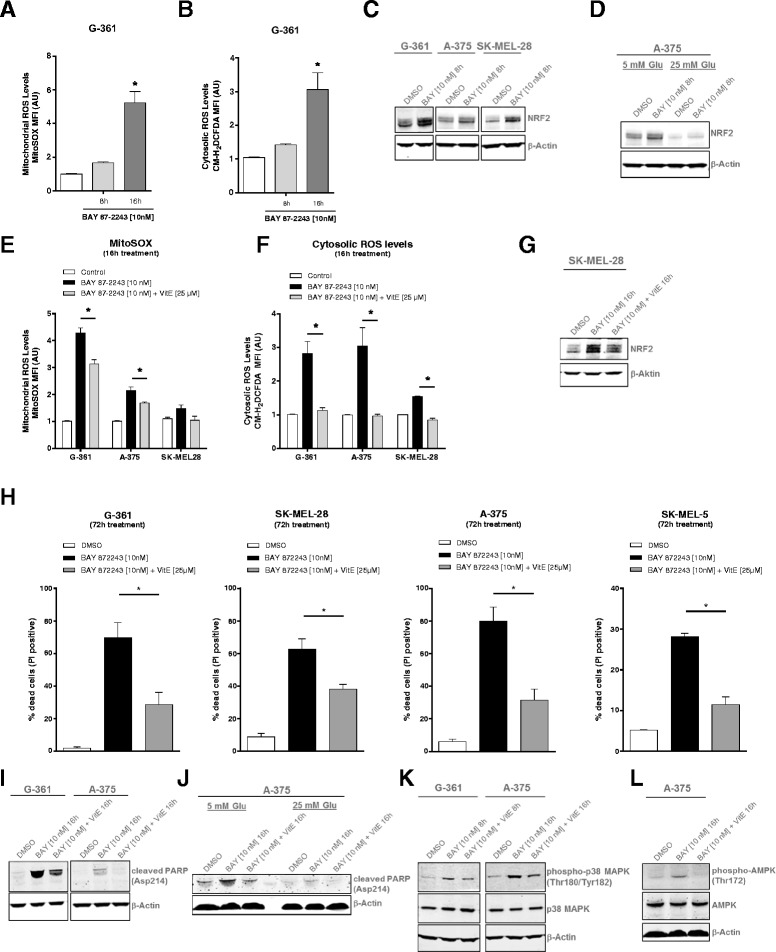
BAY 87-2243-mediated complex I inhibition increases mitochondrial and cytosolic ROS levels resulting in ROS-mediated cell death. **a** G-361 cells were treated with BAY 87-2243 (10 nM). Mitochondrial ROS levels were measured using the MitoSOX dye after 8 and 16 h (*n* = 3). **b** G-361 cells were treated with BAY 87-2243 (10 nM). Cytosolic ROS levels were measured using the ROS-reactive dye CM-H_2_DCFDA after 8 and 16 h (*n* = 3). **c**–**d** Melanoma cells were treated with BAY 87-2243 (10 nM) under (**c** and **d**) low- (5 mM) and **d** high (25 mM) glucose conditions. Cell lysates were collected from treated melanoma cell lines (8 h) and probed with antibodies recognizing NRF2. Actin was used as a loading control. **e**–**f** Melanoma cells were treated for 16 h with BAY 87-2243 (10 nM) alone or in combination with the antioxidant vitamin E (25 μM). **e** Mitochondrial and **f** cytosolic ROS levels were analyzed using flow cytometry (*n* = 3). **g** SK-MEL-28 cells were treated with BAY 87-2243 (10 nM) alone or in combination with vitamin E (25 μM). Cell lysates collected from treated SK-MEL-28 cells (16 h) were probed with antibodies recognizing NRF2. Actin was used as a loading control. **h** BRAF mutant melanoma cells were treated with BAY 87-2243 (10 nM) alone or in combination with vitamin E (25 μM). Cell death was measured after 72 h using propidium iodine (*n* = 3). **i**–**j** Melanoma cells were treated with BAY 87-2243 (10 nM) alone or in combination with vitamin E (25 μM) under (**i** and **j**) low- and **j** high glucose conditions. Cell lysates collected from treated A-375 and G-361 cells (16 h) were probed with antibodies recognizing cleaved PARP. Actin was used as a loading control. **k**–**l** BRAF mutant melanoma cells were treated with BAY 87-2243 (10 nM) alone or in combination with vitamin E (25 μM). Cell lysates collected from treated cells (8 and 16 h) were probed with antibodies recognizing **k** phospho-p38 MAPK and total p38 MAPK and **l** phospho-AMPK (Thr172) and total AMPK. Actin was used as a loading control. Data are represented as the mean ± SD. *p < 0.05

To further investigate the effect of BAY 87-2243 on ROS levels, we used the redox-reactive dye CM-H_2_DCFDA and the mitochondrial targeted dye MitoSOX which measure the levels of cytosolic and mitochondrial ROS, respectively. As expected, BAY 87-2243 generated high levels of mitochondrial ROS, in a dose-dependent manner, as well as cytosolic ROS over time in melanoma cells (Fig. 4a, b and Additional file 3: Figure S3A).

In order to evaluate the downstream effects of the BAY 87-2243-induced increase in ROS levels, we looked at the well-established ROS marker nuclear factor (erythroid-derived 2)-like 2 (NRF2), which is a transcription factor and master regulator of the antioxidant response. NRF2 is known to be stabilized upon ROS in order for the cell to balance potentially cell-damaging oxidative stress [30–32]. BAY 87-2243 induced the stabilization of NRF2 in melanoma cells (Fig. 4c), which was abrogated in the presence of high glucose (Fig. 4d). To establish whether increased levels of ROS are required for BAY 87-2243-mediated cell death, we treated melanoma cells with BAY 87-2243 in the presence of the antioxidants vitamin E and the reduced glutathione precursor *N*-acetyl cysteine (NAC). First, we show that the vitamin E and NAC reversed ROS levels upon BAY 87-2243 treatment in melanoma cells (Fig. 4e, f and Additional file 3: Figure S3B). Furthermore, NRF2 levels were reversed by the addition of vitamin E in inhibitor-treated melanoma cells (Fig. 4g). In addition to decreasing ROS stress, the antioxidant treatment abrogated BAY 87-2243-induced cell death, suggesting that ROS might play a causal role in this process (Fig. 4h and Additional file 3: Figure S3C). Our findings also translated into cell death and redox-regulated protein expression [33, 34]. The expression of the cell death marker cleaved Poly (ADP-ribose) polymerase (PARP) is induced by BAY 87-2243 and rescued in the presence of the antioxidant vitamin E and NAC (Fig. 4i and Additional file 3: Figure S3D) and also by the addition of excess glucose (Fig. 4j). Furthermore, we looked at the mitogen-activated protein kinase (MAPK) p38, which is a well characterized ROS sensor, described to induce ROS-mediated cell death [30, 35]. Indeed BAY 87-2243 treatment induced the activation of p38 MAPK which was reversed by vitamin E treatment in melanoma cells (Fig. 4k). Interestingly, BAY 87-2243-induced AMPK activation was also rescued by the antioxidant (Fig. 4l). Taken together, our data suggest that melanoma cells rely on glycolysis and the resulting ATP production, as well as on redox balance to survive in the presence of BAY 87-2243. We show that BAY 87-2243-mediated cell death is caused by ATP depletion and ROS stress, which can both be alleviated by the addition of excess glucose.

### Vemurafenib increases mitochondrial density and respiration in BRAF mutant melanoma cells

Our data show that BAY 87-2243 has substantial effects as a single agent against melanoma in vitro and in vivo, including BRAF mutant melanoma. Having established that BAY 87-2243 treatment leads to a metabolic switch away from OXPHOS making melanoma cells dependent on glycolysis, we next wanted to test whether the efficacy of BAY 87-2243 could be enhanced by a therapeutic agent, based on its mechanism of action. Recent studies have highlighted that mutant BRAF inhibition makes melanoma cells dependent on OXPHOS and mitochondrial ATP production [36]. In fact, the selective mutant BRAF^V600E^ inhibitor vemurafenib actively reduced glucose uptake in BRAF mutant melanoma cell lines [37]. In BRAF^V600E^ melanomas, this switch to OXPHOS is seen in conjunction with the development of drug resistance against selective inhibitors which commonly occurs and represents a major limitation to clinical efficacy [38, 39].

Since the mitochondrial complex I inhibitor BAY 87-2243 impairs OXPHOS and causes the cells to use glycolysis, it impinges on the dependency on mitochondrial metabolism, which is forced by BRAF inhibitors like vemurafenib. This opposing metabolic dependency suggests a rationale for combining vemurafenib with BAY 87-2243 in order to exploit the mitochondrial pathway that vemurafenib-treated melanoma cells need in order to survive. Combination treatment could potentially increase energy stress by taking away the respective adaptive metabolic upregulation leading to augmented melanoma cell death compared to single agent treatment.

**Fig. 5:**
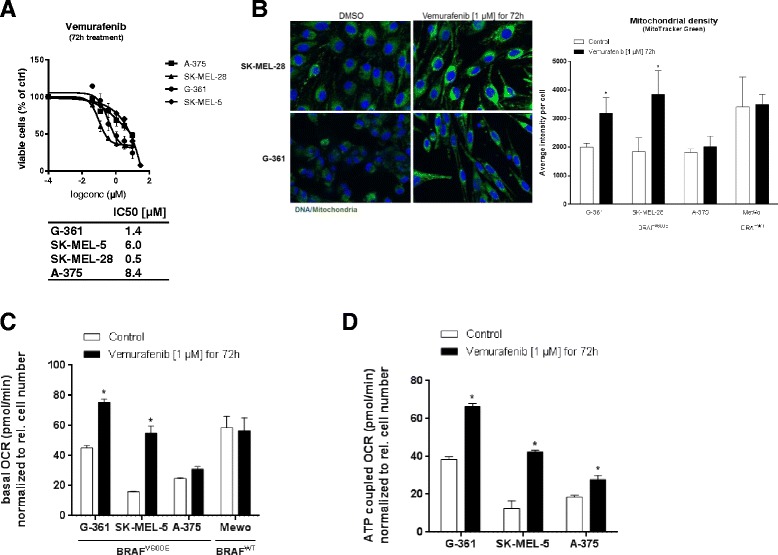
Vemurafenib increases mitochondrial density and respiration in BRAF mutant melanoma cells. **a** BRAF mutant melanoma cells were treated with various concentrations of vemurafenib. Cell proliferation was measured after 72 h. IC50 values were calculated using GraphPad prism (*n* = 4). **b** MitoTracker Green fluorescence (*left*) and quantification (*right*) (average intensity per cell) of BRAF mutant and BRAF wild type melanoma cells treated with vemurafenib (1 μM) for 72 h (*n* = 3). **c** OCR was measured using Seahorse analyzer in BRAF mutant and BRAF wild type melanoma cells after treatment with vemurafenib (1 μM) for 72 h (*n* = 3). **d** ATP-coupled OCR (after injection of oligomycin (1 μM)) was analyzed using Seahorse in BRAF mutant melanoma cells after treatment with vemurafenib (1 μM) for 72 h (*n* = 3). Data are represented as the mean ± SD. *p < 0.05

To validate the vemurafenib literature data with regard to proliferation, OXPHOS, mitochondrial function, and biogenesis, BRAF mutant melanoma cells were treated with vemurafenib for 72 h. We show that vemurafenib impaired melanoma cell proliferation (Fig. 5a) and increased mitochondrial density (Fig. 5b). The mitochondrial density of BRAF wild type cells, MeWo, was not affected by vemurafenib (Fig. 5b). Furthermore, vemurafenib led to a significant increase in basal and ATP-coupled oxygen consumption rate which was not observed in BRAF wild type cells (Fig. 5c, d). Our data suggests that vemurafenib induces OXPHOS in BRAF mutant melanoma cells, making it a good combination drug for BAY 87-2243.

### Inhibition of complex I using BAY 87-2243 in combination with vemurafenib attenuated BRAF mutant melanoma tumor growth in vivo

**Fig. 6:**
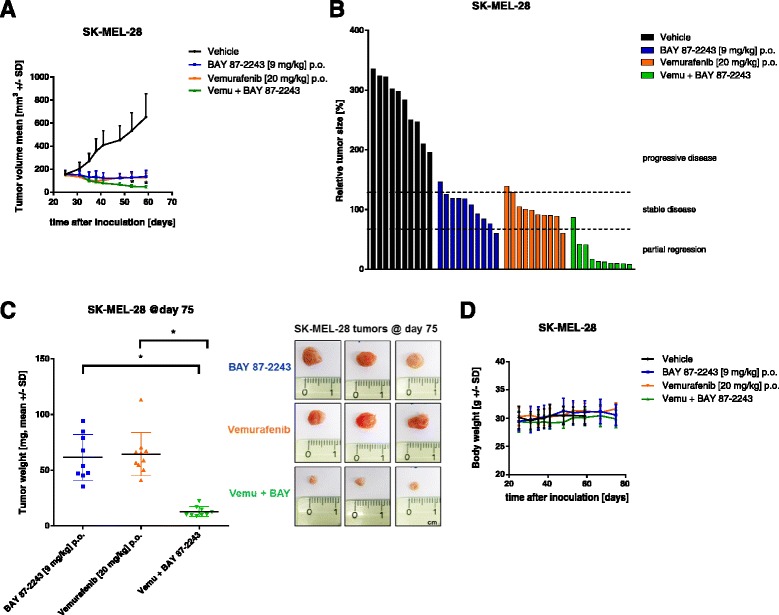
Inhibition of complex I using BAY 87-2243 in combination with vemurafenib attenuated melanoma tumor growth in vivo. **a** Nude mice were xenotransplanted with 3 × 10^6^ SK-MEL-28 (50 % matrigel) human melanoma cells (*n* = 10 per group). Mice were treated orally (p.o.) with vemurafenib (20 mg/kg, twice a day) or for combination studies with vemurafenib (20 mg/kg, twice a day) and BAY 87-2243 (9 mg/kg, once a day). Tumor volume was measured. **b** Relative tumor size (%) of individual SK-MEL-28 xenograft tumors (tumor area at study end × 100 / tumor area at study begin). Partial regression, <70 % (tumor size at study end > 30 % smaller than original tumor size at study start); stable disease, 70–120 % (tumor size between 30 % smaller and 20 % bigger of original size); progressive disease, >120 % (tumor size at study end > 20 % bigger than original size). **c** Tumor weights of individual SK-MEL-28 xenograft tumors. **d** Body weights of mice (*n* = 10) bearing human SK-MEL-28 xenografts treated orally (p.o.) once daily with vehicle (ethanol/solutol/water = 10:40:50), or with vemurafenib (20 mg/kg, twice a day), or for combination studies with vemurafenib (20 mg/kg, twice a day) and BAY 87-2243 (9 mg/kg, once a day). Data are represented as the mean ± SD. *p < 0.05

In order to test the in vivo efficacy of BAY 87-2243 and vemurafenib as single agents and in combination, we used the inhibitors in a BRAF mutant melanoma cell xenograft model. Upon subcutaneous injection of SK-MEL-28 melanoma cell, tumors were allowed to grow to around 50 mm^2^ before the mice were treated with either vehicle, 9 mg/kg BAY 87-2243, 20 mg/kg vemurafenib, or a combination of both inhibitors by oral gavage. We show that mice treated with the combination of BAY 87-2243 and vemurafenib have enhanced reduction in melanoma tumor growth compared to the reduction seen in mice treated with each agent alone (Fig. 6a, b). Furthermore, combination treatment augmented the effect on reduction in tumor weight compared to single agent treatment without affecting body weight of the mice (Fig. 6c, d). Our data indicate that BAY 87-2243-mediated complex I inhibition, in addition to its function as a single agent, may have therapeutic benefits in combination treatment with the mutant BRAF inhibitor vemurafenib in melanoma.

## Discussion

### Selective inhibition of mitochondrial complex I with BAY 87-2243 in melanoma cells induces cell death in vitro and reduces tumor growth in vivo

In recent years, mitochondrial metabolism has emerged as a target for cancer therapy due to the appreciation of mitochondria as a central metabolic organelle required for tumorigenesis [12, 19, 40]. However, there is still a lack of selective cancer therapies targeting OXPHOS and mitochondrial metabolism. In this study, we used the mitochondrial complex I small molecule inhibitor BAY 87-2243 and investigated its effects in melanoma cells. In addition to causing proliferation impairment and cell death in a variety of BRAF mutant and wild type melanoma cells, BAY 87-2243 also revealed strong anti-tumor activity as a single agent in vivo. Interestingly, BAY 87-2243 reduced tumor growth most effectively in slowly proliferating tumors (e.g., the SK-MEL-28 xenograft model) when compared to rapidly proliferating tumors (e.g., the A-375 xenograft model) (Fig. 1d and Additional file 1: Figure S1). This observation might be explained by the fact that fast proliferating tumors are more glycolytic therefore consume more glucose than slow-growing tumors [41–43]. Hence, these latter tumors are more OXPHOS-dependent and therefore more sensitive to BAY 87-2243-induced complex I inhibition. Based on our in vivo data, it is tempting to speculate that tumor growth rate might be used as a biomarker readout of sensitivity to complex I inhibition.

In line with our observation that glycolytic tumors are more resistant to complex I inhibition in vivo we suggest that BAY 87-2243 might be more effective in conditions of limiting glucose availability (e.g., in the center of a tumor). Indeed, BAY 87-2243 did not affect melanoma cell viability when 25 mM of extracellular glucose was present in the cell culture media, whereas at 5 mM glucose, the inhibitor induced ATP depletion and melanoma cell death (Fig. 3). This suggests that BAY 87-2243 targets OXPHOS in melanoma cells and that the effects thereof depend on the amount of extracellular glucose (Additional file 4: Figure S4).

Several retrospective studies suggested that metformin, commonly used in patients with type II diabetes, inhibits complex I and shows anti-tumor effects [17–21]. In contrast to metformin, BAY 87-2243 showed strong anti-proliferative effects at low nanomolar concentrations and anti-tumor effects at lower doses. Furthermore, the efficacy of metformin may be further limited by the expression of organic cation transporters (OCTs) required for uptake and subsequent inhibition of OXPHOS [44].

### BAY 87-2243 induces cell death via an oxidative stress mechanism

Compared to normal cells, cancer cells are characterized by an increased rate of ROS production. To prevent oxidative stress induction, cancer cells upregulate ROS-detoxifying systems [30, 45, 46], thereby, keeping the ROS level within its pro-tumorigenic range [14, 47]. Here, we observed that BAY 87-2243 reduced cellular ATP levels (Fig. 3a) and increased cellular ROS levels (Fig. 4a, b). This increase was associated with higher levels of NRF2 and phosphorylated p38 MAPK. Interestingly, BAY 87-2243 partially prevented ROS stimulation in 25 mM glucose medium and in the presence of antioxidants (vitamin E and NAC) (Figs. 3h, 4e, f and Additional file 3: Figure S3B). Also, under these conditions, BAY 87-2243-induced cell death was prevented (Fig. 4h and Additional file 3: Figure S3C). This suggests that BAY 87-2243 inhibits complex I and thereby stimulates ROS generation and induction of oxidative stress to induce cell death. Alternatively, a (parallel) mechanism might be active in which complex I inhibition renders the cells completely dependent on glycolysis for ATP generation. When extracellular glucose levels are limited (i.e., 5 mM), this glycolytic switch is unable to prevent ATP depletion and the ensuing cell death. Hence, BAY 87-2243 inhibits tumorigenesis through multiple mechanisms including the induction of cancer cell death in conditions where glucose concentration is limited (leading to ATP depletion) and via induction of oxidative stress and a ROS-mediated cell death (Additional file 4: Figure S4). Although it remains to be determined how high extracellular glucose levels lower ROS levels, a similar phenomenon has been observed in cells with inherited and inhibitor-induced complex I deficiency [48].

### The combination of vemurafenib and BAY 87-2243 augments anti-tumor effects in BRAF mutant melanoma

Upon treatment with the mutant BRAF^V600E^ inhibitor vemurafenib, melanoma cells upregulate mitochondrial oxygen consumption and decrease glycolysis [36]. This suggests that these cells switch their cellular ATP production from glycolysis towards mitochondrial metabolism, making their survival more dependent on mitochondrial function. In contrast, chronic inhibition of complex I was demonstrated to induce a fully glycolytic phenotype in primary human skin fibroblasts [49]. Since the latter is very similar to the effects induced by BAY 87-2243 in this study, we treated melanoma cells with a combination of vemurafenib and BAY 87-2243. Under these conditions, we expected that the vemurafenib-induced stimulation of mitochondrial metabolism is counterbalanced by the inhibition of mitochondrial function using BAY 87-2243. Interestingly, when compared to their use as single agents, the combination of BAY 87-2243 and vemurafenib induced augmented tumor regression in nude mice bearing BRAF mutant melanoma xenografts (Fig. 6). In agreement with our approach, additive effects between biguanides and BRAF inhibitor vemurafenib have been demonstrated previously [50, 51].

One explanation for the augmented anti-tumor effect might involve the activating effect of BAY 87-2243 on AMPK. It has been demonstrated that mutated BRAF^V600E^ suppresses the activity of AMPK by promoting inhibitory phosphorylation of its upstream kinase (LKB1) by ERK1/2 and that this AMPK inhibition is critical for melanoma cell proliferation and anchorage-independent growth [52]. Interestingly, we show that BAY 87-2243 suppresses the phosphorylation of ERK1/2 possibly allowing AMPK activation. Our results suggest that BAY 87-2243 induces an energy crisis in BRAF mutant melanoma cell lines accompanied by the downregulation of the RAF-MEK-ERK signaling cascade, thereby, potentially allowing activation of AMPK and RAPTOR (Fig. 3a, b, c, d, and Additional file 4: Figure S4).

Another possible explanation for the augmented anti-tumor effect of vemurafenib and BAY 87-2243 might be the heterogeneous nature of tumors with respect to oxygen availability. Relative to normal tissue, tumors display a much steeper oxygen gradient with its level dropping close to zero in necrotic core areas. Adaptation to these hypoxic conditions is for instance mediated through the stabilization of the transcription factor HIF-1, which controls the expression of many genes involved in glycolysis [53]. In this context, it was demonstrated that BAY 87-2243 treatment suppressed HIF-1α protein levels and the expression of HIF-1 target genes in a H460 xenograft model [25]. This means that BAY 87-2243 might be more effective when combined with other treatments that lower the availability of oxygen and extracellular glucose in cancer cells. Interestingly, vemurafenib has been shown to suppress glucose uptake in melanoma cells [37] and also in tumors from patients with BRAF^V600E^ melanoma [54]. Therefore, it can be hypothesized that a therapeutic advantage is achieved in BRAF mutant melanoma tumors by first blocking OXPHOS and adaptation to hypoxia with BAY 87-2243, followed by subsequent downregulating of glycolysis using vemurafenib.

## Conclusions

Compared to standard chemotherapy, vemurafenib extends melanoma survival up to 6 months [55, 56]. In spite of dramatic initial tumor shrinkage in patients with BRAF^V600E^ melanoma, long-term efficacy is thwarted due to emergence of drug resistance and drastic tumor regrowth [57]. Here, we confirm previous findings that melanoma cells adapt to BRAF inhibitors by upregulating OXPHOS therefore validating our approach to effectively target this pathway with the selective mitochondrial complex I inhibitor BAY 87-2243. Our study holds promise for the design of future combination therapies by broadening the understanding of how to prevent the development of resistance to oncogene-targeted therapies which may increase their initial therapeutic efficacy. Based on our findings, we suggest combining vemurafenib with complex I inhibitors in BRAF mutant melanoma in order to diminish the ability of cancer cells to adapt to either agent.

## Additional files

Additional file 1: Figure S1. BAY 87-2243 reduces tumor growth in melanoma cells in vivo. (A) Scid mice bearing established A-375 (1.5x10^6^ cells/mouse in 50 % matrigel, n = 10 per group) and LOX-IMVI (1.5 × 10^6^ cells/mouse in 50 % matrigel, *n* = 10 per group) human xenograft tumors were treated orally (p.o.), once daily with vehicle (Ethanol/Solutol/Water = 10:40:50) or BAY 87-2243 (9 mg/kg). (B) Tumor weights of human melanoma xenografts (A-375, G-361, SK-MEL-28, LOX-IMVI) treated orally (p.o.), once daily with vehicle (Ethanol/Solutol/Water = 10:40:50) or BAY 87-2243 (9 mg/kg). (C) Body weights of mice bearing patient-derived (MEXF 276, MEXF 1732) and human melanoma xenografts (G-361 and SK-MEL-28) treated orally (p.o.), once daily with vehicle (Ethanol/Solutol/Water = 10:40:50) or BAY 87-2243 (9 mg/kg). Data are represented as the mean ± SD. **p* < 0.05. Additional file 2: Figure S2. Inhibition of mitochondrial complex I with BAY 87-2243 inhibits OXPHOS and triggers glycolysis in melanoma cells. (A) OCR was measured using polarographic oxygen sensors in a two-chamber Oxygraph (Oroboros) in melanoma cells. BAY 87-2243 was injected in different concentrations (*n* = 3). (B) SK-MEL-28 and G-361 cells were treated with BAY 87-2243 (10 nM) and the time-dependent production of extracellular lactate was measured (*n* = 4). (C) OCR was measured using Seahorse analyzer in G-361 cells. BAY 87-2243 was injected (black arrow) in different concentrations followed by consecutive injections of oligomycin (1 μM), FCCP (0.5 μM) and antimycin A (1 μM)/rotenone (1 μM) (*n* = 6). Data are represented as the mean ± SD. **p* < 0.05. Additional file 3: Figure S3. Complex I inhibition causes oxidative stress and ROS-mediated cell death. (A) G-361 and SK-MEL-28 cells were treated with increasing concentrations of BAY 87-2243. Mitochondrial ROS levels were measured using the MitoSOX dye after 24 hours (*n* = 2). (B) A-375 cells were treated with BAY 87-2243 (10 nM) and the antioxidant, NAC (5 mM). Cytosolic ROS levels were measured using the redox-reactive dye CM-H_2_DCFDA after 24 h (*n* = 3). (C) Melanoma cells were treated with BAY 87-2243 (10 nM) and the antioxidant NAC (5 mM). Cell death was measured after 72 h using propidium iodine (*n* = 3). (D) G-361 cells were treated with BAY 87-2243 (10 nM, 100 nM) and the antioxidant, NAC (5 mM). Cell lysates were collected from treated G-361 cells (24 h) and probed with antibodies recognizing cleaved PARP. Actin was used as a loading control. Data are represented as the mean ± SD. **p* < 0.05. Additional file 4: Figure S4. Melanoma cells are sensitive to BAY 87-2243-mediated Complex I inhibition by undergoing an energy crisis and ROS-mediated cell death. Under limiting glucose conditions (5 mM), the mitochondrial complex I inhibitor BAY 87-2243 induces a metabolic switch from OXPHOS to glycolysis in melanoma cells. Results of BAY 87-2243-mediated complex I inhibition include a reduction in the total cellular ATP pool and therefore AMPK activation, suppression of ERK1/2 phosphorylation, and the induction of oxidative stress. ROS stress is marked by stabilization of NRF2, phosphorylation of p38 MAPK, and AMPK as well as cell death signaling marked by cleaved PARP.

## Abbreviations

AMPK: AMP-activated protein kinase
ECAR: extracellular acidification rate
OCR: oxygen consumption rate
OXPHOS: oxidative phosphorylation
ROS: reactive oxygen species
