# Supplementary figures and images for: Targeting mitochondrial complex I using BAY 87-2243 reduces melanoma tumor growth
Source: Cancer Metab. 2015 Oct 20;3:11. doi: 10.1186/s40170-015-0138-0 (PMC4615872; doi:10.1186/s40170-015-0138-0)

## Slide 1
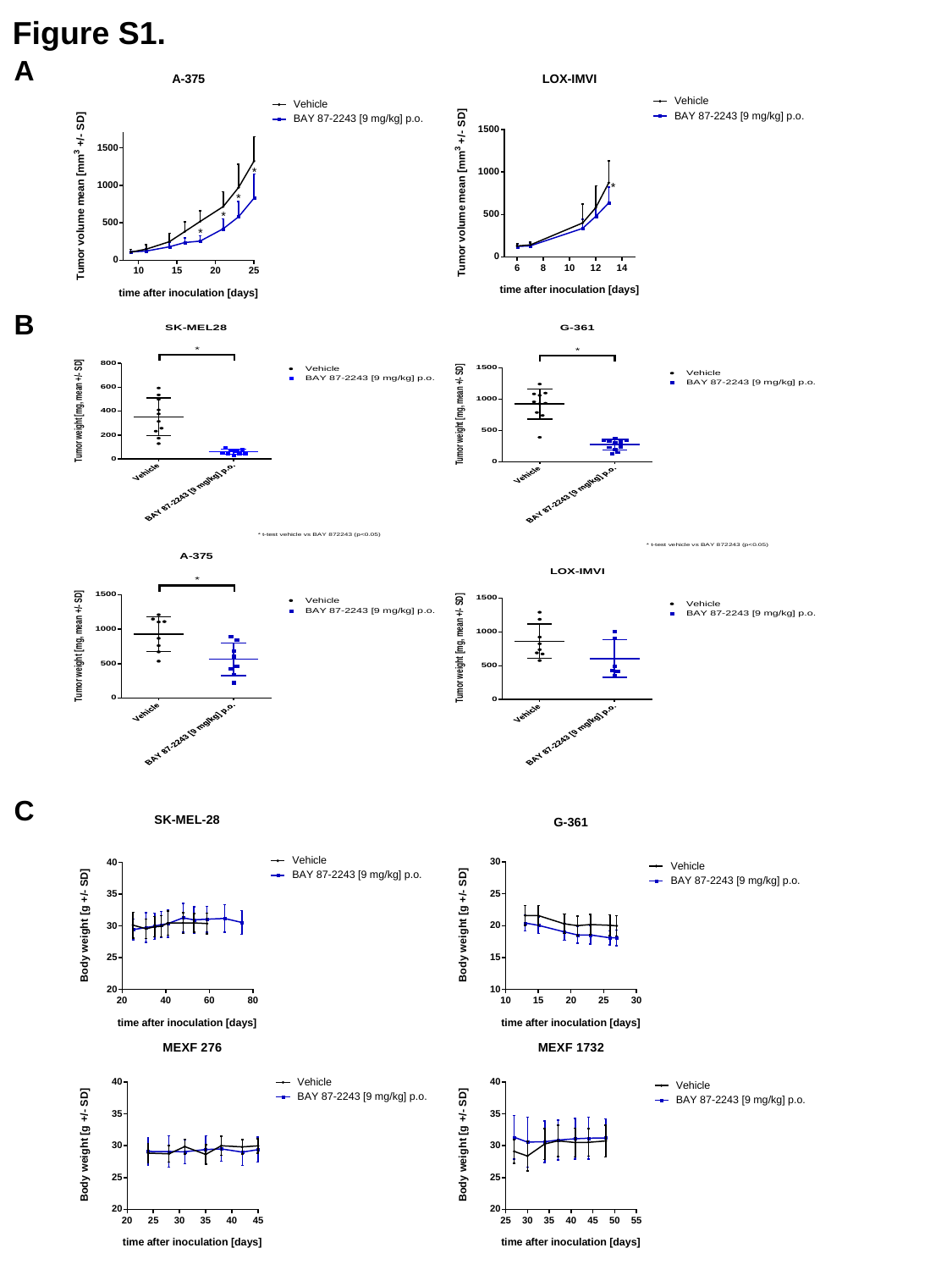

Figure S1.
A
B
C

Supplement: Additional file 1: Figure S1. — BAY 87-2243 reduces tumor growth in melanoma cells in vivo. (A) Scid mice bearing established A-375 (1.5x10^6^ cells/mouse in 50 % matrigel, n = 10 per group) and LOX-IMVI (1.5 × 10^6^ cells/mouse in 50 % matrigel, n = 10 per group) human xenograft tumors were treated orally (p.o.), once daily with vehicle (Ethanol/Solutol/Water = 10:40:50) or BAY 87-2243 (9 mg/kg). (B) Tumor weights of human melanoma xenografts (A-375, G-361, SK-MEL-28, LOX-IMVI) treated orally (p.o.), once daily with vehicle (Ethanol/Solutol/Water = 10:40:50) or BAY 87-2243 (9 mg/kg). (C) Body weights of mice bearing patient-derived (MEXF 276, MEXF 1732) and human melanoma xenografts (G-361 and SK-MEL-28) treated orally (p.o.), once daily with vehicle (Ethanol/Solutol/Water = 10:40:50) or BAY 87-2243 (9 mg/kg). Data are represented as the mean ± SD. *p < 0.05. [file 40170_2015_138_MOESM1_ESM.ppt]

## Slide 1
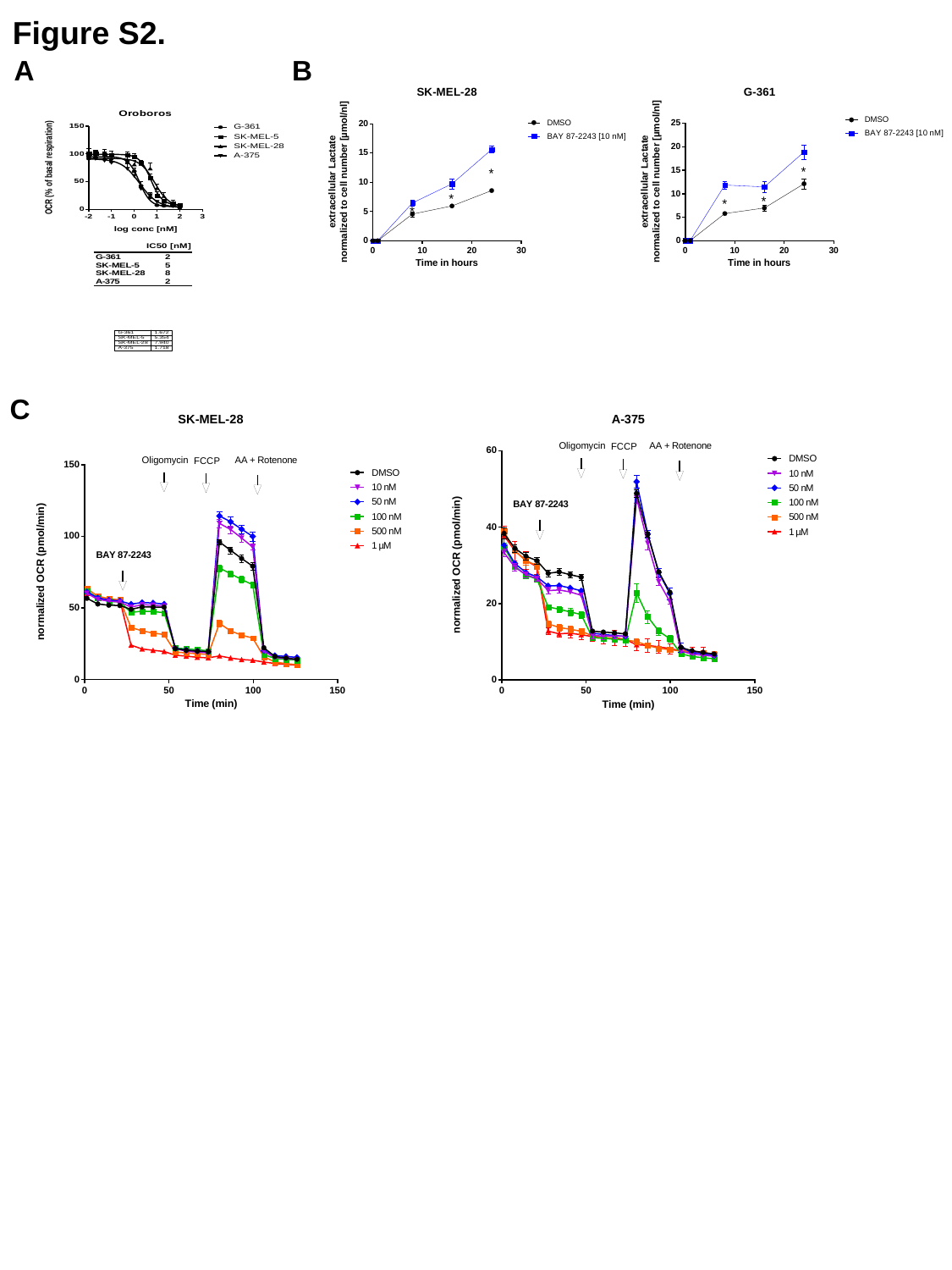

Figure S2.
A
B
C

Supplement: Additional file 2: Figure S2. — Inhibition of mitochondrial complex I with BAY 87-2243 inhibits OXPHOS and triggers glycolysis in melanoma cells. (A) OCR was measured using polarographic oxygen sensors in a two-chamber Oxygraph (Oroboros) in melanoma cells. BAY 87-2243 was injected in different concentrations (n = 3). (B) SK-MEL-28 and G-361 cells were treated with BAY 87-2243 (10 nM) and the time-dependent production of extracellular lactate was measured (n = 4). (C) OCR was measured using Seahorse analyzer in G-361 cells. BAY 87-2243 was injected (black arrow) in different concentrations followed by consecutive injections of oligomycin (1 μM), FCCP (0.5 μM) and antimycin A (1 μM)/rotenone (1 μM) (n = 6). Data are represented as the mean ± SD. *p < 0.05. [file 40170_2015_138_MOESM2_ESM.ppt]

## Slide 1
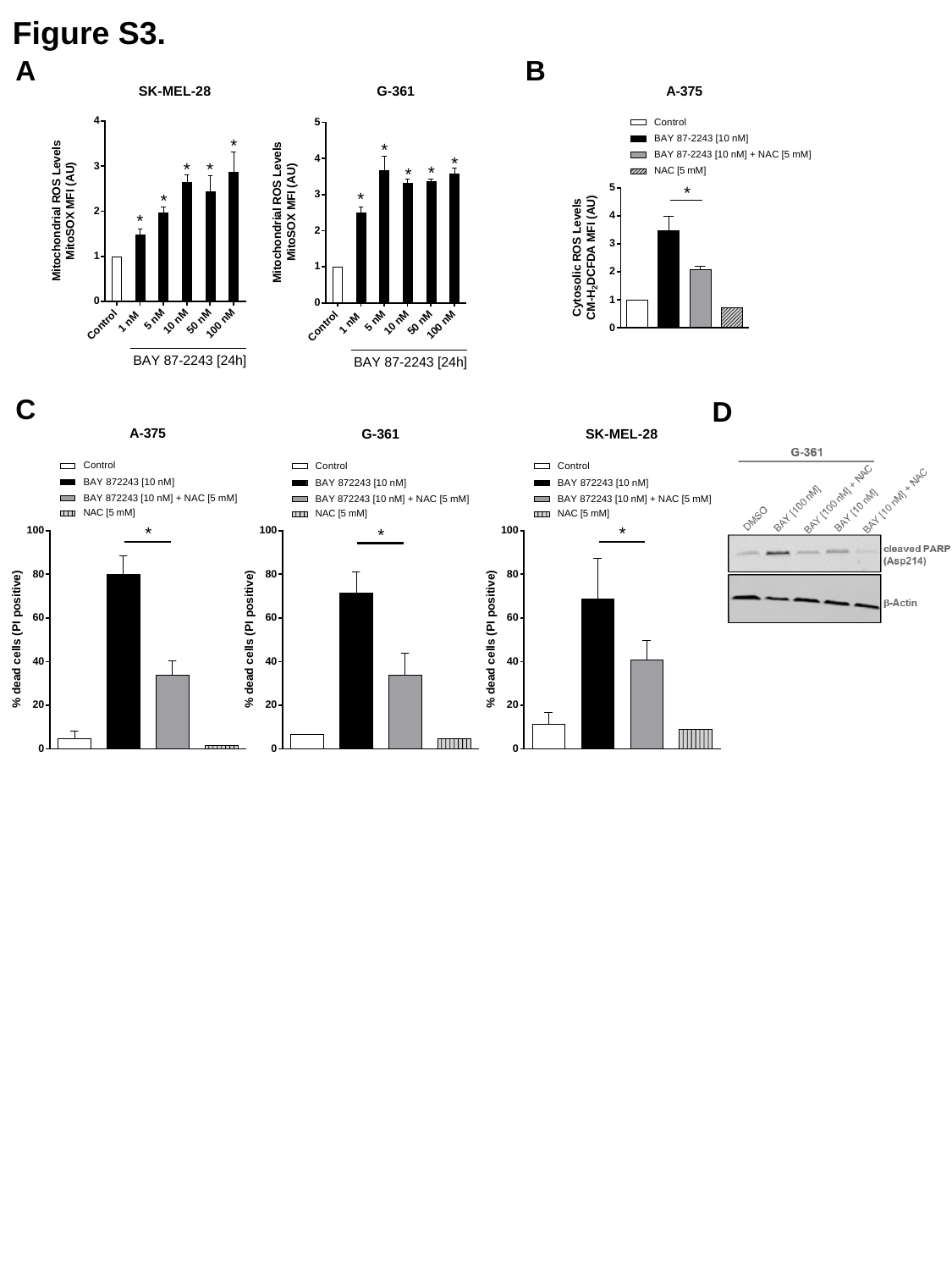

Figure S3.
A
B
C
D

Supplement: Additional file 3: Figure S3. — Complex I inhibition causes oxidative stress and ROS-mediated cell death. (A) G-361 and SK-MEL-28 cells were treated with increasing concentrations of BAY 87-2243. Mitochondrial ROS levels were measured using the MitoSOX dye after 24 hours (n = 2). (B) A-375 cells were treated with BAY 87-2243 (10 nM) and the antioxidant, NAC (5 mM). Cytosolic ROS levels were measured using the redox-reactive dye CM-H2DCFDA after 24 h (n = 3). (C) Melanoma cells were treated with BAY 87-2243 (10 nM) and the antioxidant NAC (5 mM). Cell death was measured after 72 h using propidium iodine (n = 3). (D) G-361 cells were treated with BAY 87-2243 (10 nM, 100 nM) and the antioxidant, NAC (5 mM). Cell lysates were collected from treated G-361 cells (24 h) and probed with antibodies recognizing cleaved PARP. Actin was used as a loading control. Data are represented as the mean ± SD. *p < 0.05. [file 40170_2015_138_MOESM3_ESM.ppt]
