# Supplementary material for: Targeting mitochondrial complex I using BAY 87-2243 reduces melanoma tumor growth
Source: Cancer Metab. 2015 Oct 20;3:11. doi: 10.1186/s40170-015-0138-0 (PMC4615872; doi:10.1186/s40170-015-0138-0)
Supplement: Additional file 4: Figure S4. — Melanoma cells are sensitive to BAY 87-2243-mediated Complex I inhibition by undergoing an energy crisis and ROS-mediated cell death. Under limiting glucose conditions (5 mM), the mitochondrial complex I inhibitor BAY 87-2243 induces a metabolic switch from OXPHOS to glycolysis in melanoma cells. Results of BAY 87-2243-mediated complex I inhibition include a reduction in the total cellular ATP pool and therefore AMPK activation, suppression of ERK1/2 phosphorylation, and the induction of oxidative stress. ROS stress is marked by stabilization of NRF2, phosphorylation of p38 MAPK, and AMPK as well as cell death signaling marked by cleaved PARP. [file 40170_2015_138_MOESM4_ESM.pptx]

## Slide 1
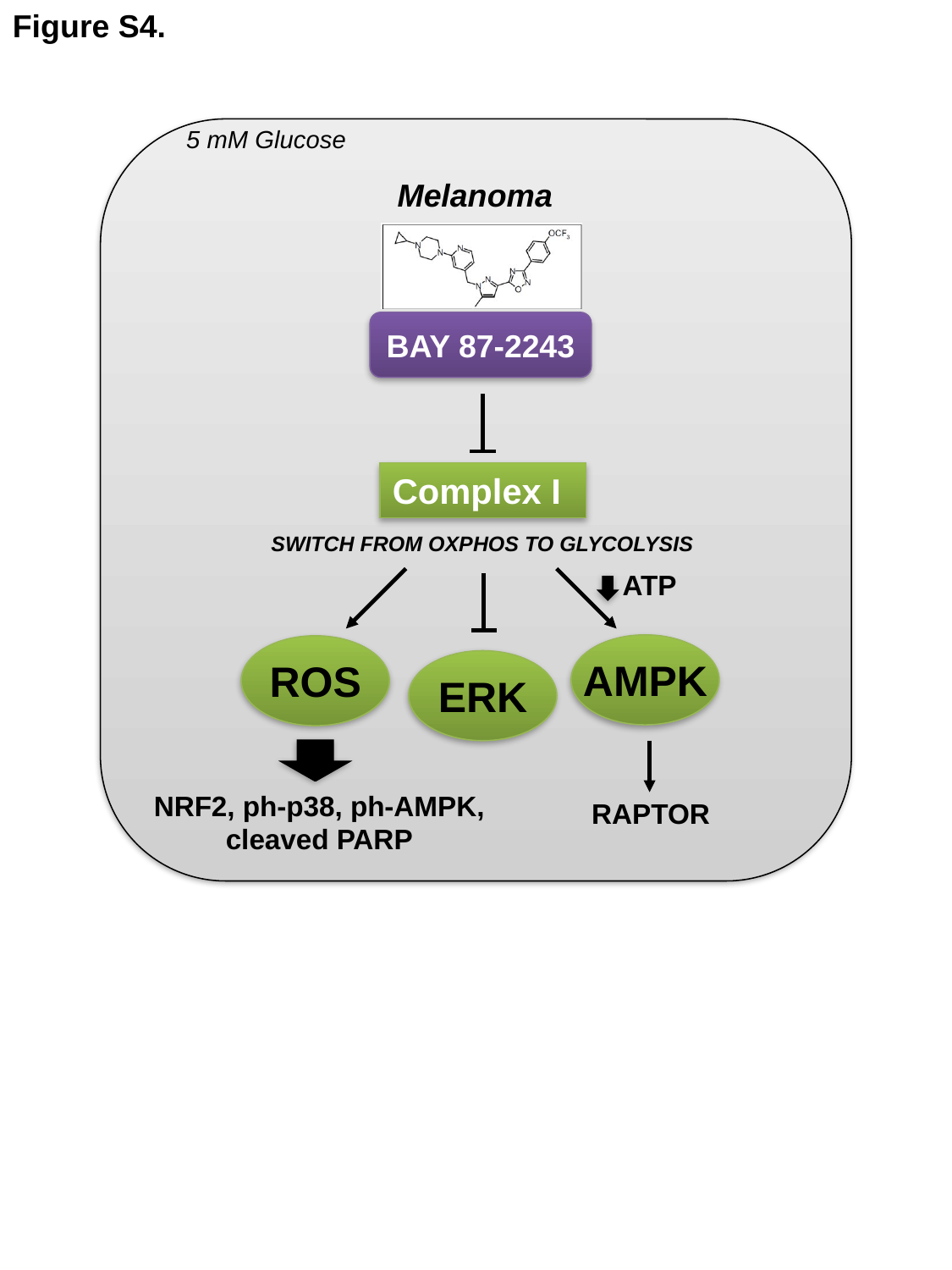

Figure S4.
5 mM Glucose
Melanoma
BAY 87-2243
Complex I
SWITCH FROM OXPHOS TO GLYCOLYSIS
ATP
AMPK
ROS
ERK
NRF2, ph-p38, ph-AMPK, cleaved PARP
RAPTOR
